# Circulating extracellular vesicle miRNA as a novel predictive marker of radiation toxicity in breast cancer patients

**DOI:** 10.3389/fonc.2026.1904861

**Published:** 2026-08-05

**Authors:** Bridget Quinn, Mina McGinn, Christopher Rabender, Douglas Arthur, Vasily Yakovlev

**Affiliations:** Massey Comprehensive Cancer Center, Virginia Commonwealth University, Richmond, VA, United States

**Keywords:** biomarkers, breast cancer, extracellular vesicles, microRNA, oxidative stress, personalized medicine, radiation-induced toxicity, radiotherapy

## Abstract

**Introduction:**

Radiotherapy is an essential component of breast cancer management; however, interpatient variability in radiation-induced normal tissue toxicity remains a significant clinical challenge. The identification of minimally invasive biomarkers capable of predicting susceptibility to radiotoxicity could facilitate personalized treatment strategies. Extracellular vesicle (EV) microRNAs (miRNAs) are stable in circulation and play key roles in cellular stress and inflammatory responses, making them attractive biomarker candidates.

**Methods:**

In this study, plasma-derived EV miRNA profiles were analyzed in breast cancer patients receiving adjuvant radiotherapy and stratified according to the severity and timing of normal tissue toxicity. EVs were isolated using a combined size-exclusion chromatography and precipitation approach and characterized by nanoparticle tracking analysis and protein marker expression. Differential miRNA expression was assessed using quantitative PCR following normalization with stably expressed endogenous controls.

**Results:**

Two EV miRNAs were associated with increased susceptibility to radiotherapy-induced toxicity. MiR-222-3p was significantly upregulated in patients who developed acute high-grade toxicity, while miR-144-5p was increased in both acute and late high-toxicity groups. Additionally, miR-200a-3p and miR-335-3p were enriched in patients exhibiting minimal or no toxicity.

**Discussion:**

These findings identify EV miRNAs with potential utility as biomarkers of radiation-induced normal tissue toxicity and support further investigation into their mechanistic roles and clinical applicability in personalized radiotherapy.

## Introduction

Breast cancer affects approximately one in eight women worldwide and remains the second leading cause of cancer-related mortality among women in the United States (1, 2). Although breast cancer mortality has declined over recent decades due to advances in early detection and treatment, it remains the most frequently diagnosed cancer in women globally (2). Moreover, standard cancer therapies often impose substantial physical toxicity and do not uniformly improve patient prognosis (3). These challenges highlight the need for improved, biologically informed treatment strategies that account for tumor-specific and patient-specific genetic differences.

Radiotherapy is a cornerstone of non-surgical cancer treatment and is administered in more than half of all breast cancer cases (3). Ionizing radiation induces cytotoxicity by exploiting the genomic instability of tumor cells, which accumulate mutations that are inefficiently repaired due to defective DNA damage response mechanisms (4). As a result, malignant cells are generally more susceptible to radiation-induced damage than normal tissues. However, healthy tissues are frequently affected as well, limiting the therapeutic window of radiotherapy (3).

Radiation-induced toxicities are commonly classified as acute or late effects. Acute toxicities typically develop during or shortly after treatment and may persist for several months, primarily affecting rapidly proliferating tissues such as the epidermis and hair follicles (3, 5). These effects include dermatitis, breast pain, and hyperpigmentation. Late toxicities may arise months to years after treatment, are often irreversible, and include fibrosis, collagen remodeling, vascular damage, and structural changes in irradiated tissues and adjacent organs (3, 5). Because of these adverse effects, radiation dose escalation is frequently constrained despite marked interpatient variability in treatment response (3). Despite the well-documented potential toxicities that come with breast irradiation, there is limited understanding of an individual patient’s sensitivity to that radiation. While most patients will experience some of the known toxicities associated with breast cancer radiation, a small number of patients will experience either very minimal or much more severe radiation toxicities. The clinical challenge, then, is sufficiently consenting a patient to a wide array of possible reactions to their treatment. It also means that Radiation Oncologists are forced to take a reactive approach to radiation toxicity as opposed to a proactive one. Having knowledge upfront of a particular patient’s radiation sensitivity would allow Physicians to not only plan their treatment in a way that may reduce toxicity but also take steps to prophylactically manage expected higher grade side effects. The ability to predict an individual’s toxicity response to breast radiation would be an incredible step forward in creating more tailored and less toxic breast cancer therapy. Although several candidate genes have been implicated in radiosensitivity, reliable biomarkers capable of predicting individual responses to radiotherapy are currently lacking (6, 7).

MicroRNAs (miRNAs) have emerged as key regulators of gene expression and represent a rapidly expanding area of cancer research. MiRNAs are short, non-coding RNA molecules (18–25 nucleotides) that regulate post-transcriptional gene expression by binding to complementary sequences in target messenger RNAs, leading to mRNA destabilization or translational repression (8, 9). Beyond their intracellular functions, miRNAs can mediate intercellular communication and influence cellular behavior in both local and distant tissues (10). They are detectable in a wide range of biological fluids, including plasma, serum, saliva, urine, and breast milk, either freely associated with RNA-binding proteins such as AGO2 or encapsulated within extracellular vesicles (EVs) (8, 9, 11). More than 2,000 mature miRNAs have been identified in humans, many of which are dysregulated in cancer and contribute to tumor growth, invasion, and metastasis (10).

In breast cancer, non-coding RNAs, including miRNAs and long non-coding RNAs (lncRNAs), regulate multiple signaling pathways involved in tumor initiation, progression, therapeutic response, and endocrine resistance (12, 13). Their complex interactions with key molecular pathways have generated considerable interest in the development of RNA-based biomarkers and therapeutic strategies for precision oncology. Nevertheless, comparatively little is known about the contribution of circulating EV-associated miRNAs to interpatient variability in radiation-induced normal tissue toxicity.

EVs have garnered particular interest due to their role in transporting miRNAs and other bioactive cargo between cells. They originate from the endosomal pathway, where intraluminal vesicles form within multivesicular bodies through mechanisms involving the ESCRT machinery (14, 15). Selective miRNA loading into EVs occurs through both passive and active mechanisms, including recognition of specific sequence motifs by RNA-binding proteins (10). Following secretion, EVs interact with recipient cells through multiple pathways, including receptor-ligand interactions, membrane fusion, and endocytosis, ultimately delivering their cargo and modulating cellular function (14, 16, 17).

The clinical potential of EV-associated miRNAs continues to expand as their biogenesis, targeting, and functional roles become better understood. EV-associated miRNA profiling has shown promise for cancer detection, monitoring disease progression, and characterizing tumor molecular profiles (18). Furthermore, therapeutic strategies aimed at inhibiting oncogenic miRNAs or restoring tumor-suppressive miRNAs are actively being explored (9, 11).

Despite this progress, the role of miRNAs – particularly EV-associated miRNAs – in determining radiosensitivity and radiation-induced toxicity remains relatively underexplored. Tumor heterogeneity contributes to variable radiotherapy responses, yet accurately predicting this variability remains challenging (18). Prior studies have identified miRNAs involved in tumor radiosensitivity and radiation response pathways, including miRNAs associated with DNA damage repair, oxidative stress, and apoptosis (19, 20). However, investigations into miRNAs associated with normal tissue toxicity are limited. A small number of studies have implicated specific miRNAs in radiation-induced cardiovascular and tissue toxicities, but these analyses have largely focused on total circulating miRNAs rather than those encapsulated within EVs (6, 21). This gap underscores the need for studies specifically examining EV-associated miRNA expression in the context of radiotherapy-induced toxicity in breast cancer patients.

The present study aims to identify EV-associated miRNAs whose baseline expression may predict individual susceptibility to radiation-induced toxicity in normal tissues. By analyzing plasma-derived EV-associated miRNAs from breast cancer patients with varying degrees of radiotoxicity, this study seeks to identify reliable biomarkers that can inform personalized radiotherapy strategies. Using quantitative PCR as a high-throughput screening approach, this work evaluates the differential expression of hundreds of known miRNAs. The identified miRNAs may not only serve as predictive biomarkers but also provide mechanistic insight into the molecular pathways governing radiation response, ultimately contributing to improved risk stratification and individualized patient care.

## Methods

### Participants and blood samples

The patients included in this study were enrolled in MCC-12181, an Institutional Review Board (IRB)-approved prospective clinical trial that collected blood samples from patients undergoing radiation therapy for cancer. Written informed consent was obtained from all participants. According to the study protocol, blood samples were collected one day before the initiation of radiation therapy and at multiple time points thereafter. For the present analysis, only patients with breast cancer who underwent surgical resection of the primary tumor followed by adjuvant radiotherapy were included. No chemotherapy, endocrine therapy, targeted therapy, or other treatment had been administered prior to sample collection. Patients underwent either breast-conserving surgery or mastectomy with or without reconstruction. Radiation techniques included both external beam and brachytherapy approaches. All of the blood samples analyzed in this study were collected 1 day prior to the patient starting radiation. Blood samples collected at additional time points were not included in the presented data. In total, samples from 89 patients were included in the analysis.

Clinical outcomes, including acute and long-term toxicities, were evaluated and data collected for those patients for whom we had blood samples. Patients were divided into four groups based on their observed radiation side effects. Group 1 contained 9 patients who had very minimal to virtually no side effects from breast radiation. Group 2 contained 45 patients who had an expected level of appropriate side effects from radiation (Grade 1- mild Grade 2 toxicities). Group 3 contained 7 patients who had higher-grade acute toxicities with subsequent resolution (Grade 2–3 dermatitis, edema, pigmentation changes), while Group 4 had 28 patients with higher-grade long-term toxicity (Grade 2–3 fibrosis/fat necrosis/pigmentation changes). For the purposes of this study, possible toxicities meeting criteria for inclusion included acute radiation dermatitis, fibrosis, hyperpigmentation and fat necrosis. Toxicity grading was based on CTCAE version 5 criteria.

The blood samples were collected in the tubes coated on the inside with K2EDTA (Potassium Ethylene Diamine Tetra Acetic acid). On the same day of collection, blood samples were centrifuged at 3,000x g for 25 minutes at 4 °C. After centrifugation plasma part of all samples was aliquoted and saved in vapor phase of liquid nitrogen.

### EVs isolation

**Figure 1:**
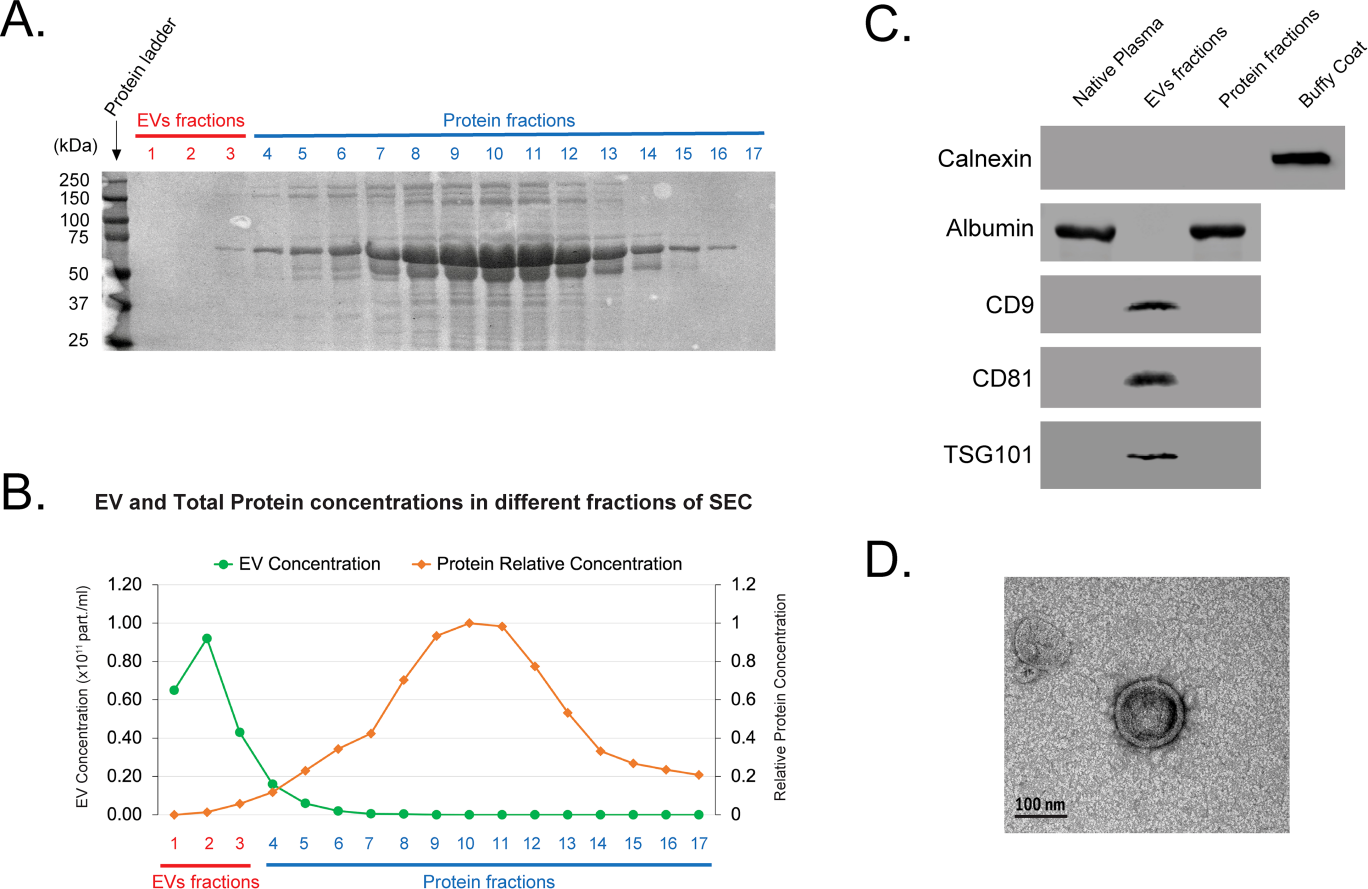
Analysis of plasma EVs after extraction by SEC. **(A)** Ponceau S staining of the different plasma fractions after extraction of EVs from plasma by SEC. **(B)** Concentrations of EVs and total protein in different plasma fractions (as shown in section A) after extraction of EVs by the SEC. **(C)** Western blot analysis of the EV-specific protein markers and negative controls. Equal amount of total protein was loaded for native plasma, EV fractions, protein fractions, and buffy coat. **(D)** Electron microscopy analysis for the extracted EVs. Scale bar = 100 nm.

EVs were extracted from plasma samples by a two-step method. The size exclusion chromatography (SEC) method using the qEVoriginal columns (Izon Science) was applied as a first step. Prior to EV isolation, SEC columns were conditioned by washing with freshly filtered (0.1 µm) phosphate-buffered saline (PBS). To remove cell debris, platelets, apoptotic bodies and other large vesicles, thawed plasma was filtered (0.2 µm). After filtration 0.5 ml of plasma samples were added to the SEC sample reservoir, and EVs were eluted in PBS, which was added to the sample reservoir as the last of the plasma entered the column. During EV isolation, the volume of PBS in the reservoir was kept below 2 mL. The initial fractions (void volume) of flow-through were discarded, and EVs were collected as pooled fractions 1–3 (1.5 mL total) (**Figures 1A, B**). Protein fractions of plasma were also collected as pooled fractions 7–13 (3.5 mL total) and stored at −20 °C. Every 0.5 ml of plasma yielded 1.5 ml of purified EVs in the filtered PBS buffer. This method has been described in detail previously (22). As the second step, EVs were precipitated using the PureExo^®^ Exosome Isolation Kit for Serum and Plasma (101Bio, Palo Alto, CA, USA) according to the manufacturer’s instructions. Precipitated EVs were reconstituted in 0.25 ml of the filtered (100 nm filter) 1xPBS buffer. Following the two-step extraction procedure, the reconstituted EVs were subjected to downstream characterization by Western blotting, nanoparticle tracking analysis, and transmission electron microscopy, as well as to RNA extraction for subsequent molecular analyses.

### Western blotting and antibodies

Equal amount of the total protein from each sample was loaded and separated by SDS-PAGE gel and transferred to nitrocellulose membranes. The membranes were exposed to antibodies at specific dilutions. Primary antibodies used for WB: anti-CD9 (dilution 1:1000, Novus Biologicals), anti-CD81 (dilution 1:1000, Cell Signaling), anti-TSG101 (dilution 1:500, Cell Signaling), anti-Albumin and anti-Calnexin (both - dilution 1:1000, Cell Signaling). Specific protein bands were detected using infrared-emitting conjugated secondary antibodies: anti-rabbit DyLight™ 800 4X PEG Conjugate (dilution 1:10,000, Cell Signaling). WB images were acquired using the Odyssey CLx Near-infrared Imaging System (LI-COR Biosciences) and analyzed with Image Studio software (version 6.1, LI-COR Biosciences).

### Nanoparticle tracking analysis

The concentration and size of the extracted EVs were measured using ZetaView^®^ Nanoparticle Tracking Analysis. All samples were diluted in filtered PBS to a final volume of 2 ml. Ideal measurement concentrations were found by pre-testing the ideal particle per frame value (140–200 particles/frame). The manufacturer’s default software settings for plasma EVs were selected accordingly. For each measurement, three cycles were performed by scanning 11 cell positions each and capturing 80 frames per position under following settings: Focus: autofocus; Camera sensitivity for all samples: 78; Shutter: 100; Scattering Intensity: detected automatically; Cell temperature: 25 °C. After capture, the videos were analyzed by the in-built ZetaView Software 8.04.02 SP2 with specific analysis parameters: Maximum area: 1000, Minimum area 5, Minimum brightness: 25. Hardware: embedded laser: 40 mW at 488 nm; camera: CMOS. The number of completed tracks in NTA measurements was always greater than the proposed minimum of 1000 to minimize data skewing based on single large particles.

### Transmission electron microscopy

Isolated EVs were fixed and prepared for the TEM as was previously described (23). The TEM analysis was carried out by the Microscopy Core of VCU.

### EV-associated RNA isolation and RT-PCR

Total RNA was isolated from the EV samples following the manufacturer’s instructions with the miRNeasy MicroRNA Extraction Kit (Qiagen) and then converted to cDNA using the miRCURY LNA RT Kit (Qiagen). The samples with total EV RNA and cDNA were stored in a freezer at −80 °C. The concentration and purity of the EV RNA samples were estimated using NanoDrop ND-1000 spectrophotometer (Thermo Scientific, Wilmington DE).

All cDNA samples were pooled into four groups (clinical Groups 1-4) and then assayed using the Serum/Plasma Focus PCR panel (Qiagen). This commercially available microarray platform contains 179 LNA miRNA primer sets of miRNAs that have been commonly found in human plasma. The Serum/Plasma Focus miRNA PCR Panels include potential reference genes and probes for evaluating potential sample contamination or damage (e.g., hemolysis). Each PCR panel also contains set of the negative control (H_2_O) and five sets of RNA Spike-In controls to evaluate RNA extraction (Spike-Ins 2-4-5; concentration ratio 1:100:10000) and cDNA synthesis (Spike-In 6) and to perform inter-plate calibration (Spike-In 3). The amplification was performed in a QuantStudio 5 Real-Time PCR System (Thermo Fisher Scientific, USA). Samples were amplified using RT2 SYBR^®^ Green qPCR Mastermix with ROX (carboxy-X-rhodamine) passive reference dye from QIAGEN. The real-time PCR data were normalized by ROX passive reference. Amplification curves were analyzed using QuantStudio™ Design & Analysis Software v1.4.2 to determine Ct values and assess melting curves. All assays exhibited a single, specific melting peak with Tm values within the manufacturer’s specifications. Relative miRNA expression levels were calculated using the comparative Ct (2^−ΔΔCt) method, with Ct values normalized to the selected reference miRNA and expressed as fold change relative to the control group.

### RT-PCR data analysis

The amplification curves were analyzed using the QuantStudio™ Design & Analysis Software v1.4.2, both for determination of Ct values and for melting curve analysis. All assays were inspected for distinct melting curves (**Supplementary Figure 1**) and the Tm was checked to be within known specifications for each particular assay. Potential reference genes were identified by comparing the overall stability and relative expression of candidate miRNAs across all groups.

### Statistical analysis

Statistical analyses were performed using GraphPad Prism (version 11; GraphPad Software, San Diego, CA, USA). Continuous variables are presented as mean values with ranges, and categorical variables are presented as frequencies and percentages. Differences in demographic and clinical characteristics among the four patient groups were assessed using the Kruskal–Wallis test for continuous variables and Pearson’s chi-square (χ^2^) or Fisher’s exact test for categorical variables. Relative expression levels of extracellular vesicle (EV)-associated miRNAs were calculated using the 2^-ΔΔCt method and compared among the four patient groups using the Kruskal–Wallis test followed by Dunn’s multiple-comparisons test. Statistical analyses were performed using the original 2^−ΔΔCt values. All statistical tests were two-sided, and p-values < 0.05 were considered statistically significant. MiRNA expression data are presented as Medians and Interquartile Ranges (IQR).

## Results

**Table 1** provides a summary of the clinical characteristics of patients in the four study groups. Among the 89 participants included in this study, 9 (10.1%) exhibited minimal or no radiotherapy (RT) – associated toxicity, 45 (52.9%) experienced expected RT-associated toxicity, 7 (7.9%) developed acute Garde 2–3 RT-associated toxicity, and 28 (31.5%) demonstrated late-onset Grade 2–3 RT-associated toxicity.

**Table 1:** Demographic and clinical features of the breast cancer patients’ groups.

Characteristic	Group 1	Group 2	Group 3	Group 4	p-value
Number of participants	9	45	7	28	–
Age (mean, range), years	54.8 (48.0-66.5)	57.1 (39.9-81.6)	49.3 (44.4-54.5)	62.5 (45.7-83.2)	0.004
Race:					
White	4	29	2	15	0.26
Black	5	16	5	13	
Grade:					
1	0	12	2	8	0.32
2	3	21	2	11	
3	6	12	3	9	
ER/PR:					
Positive	8	41	6	25	0.97
Negative	1	4	1	3	
HER2:					
Positive	2	8	2	2	0.41
Negative	7	37	5	26	
Adjuvant hormonal Tx:					
Yes	8	34	4	22	0.51
No	1	11	3	6	

Group 1: very minimal to no side effects.

Group 2: expected level of appropriate side (Grade 1, mild Grade 2).

Group 3: higher-grade acute toxicity with subsequent resolution (Grade 2-3).

Group 4: higher-grade long-term toxicity (Grade 2-3).

### Extraction of EVs from plasma samples

**Figure 2:**
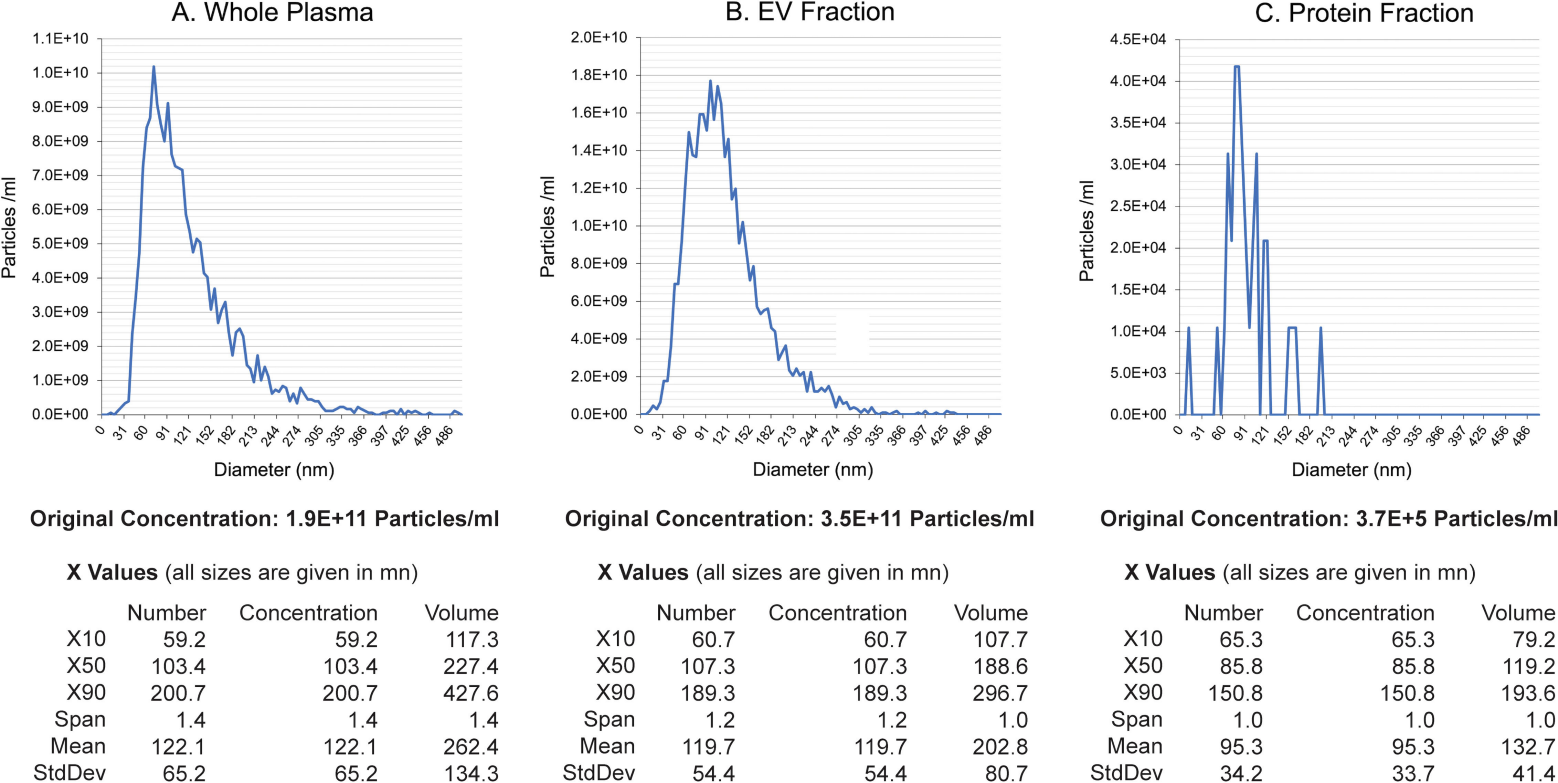
Nanoparticle tracking analysis (ZetaView^®^) of EV concentration and size representation (The figure represents the profile of a single representative patient). EVs were extracted from 0.5 ml of plasma by SEC and precipitated from 1.5 ml of EVs fractions. Precipitated EVs were reconstituted in 0.25 ml of 1X PBS buffer. **(A)** the whole filtered plasma before EVs isolation; **(B)** EVs reconstituted after SEC extraction and precipitation; **(C)** Combined protein fractions #7-13 after SEC.

The quality and quantity of plasma-derived EVs were assessed using multiple analytical approaches. Western blot analysis demonstrated that, following SEC, EV-specific protein markers CD9, CD81, and TSG101 were detected exclusively in the EV fraction (**Figure 1C**). In contrast, this fraction showed no detectable contamination with Albumin, the most abundant plasma protein, and Calnexin, an endoplasmic reticulum-associated protein, serves as a negative marker for extracellular vesicle preparations and indicates contamination with intracellular organelles, apoptotic bodies, cell debris, or material released as a result of extensive cellular lysis during sample processing (**Figure 1C**). Transmission electron microscopy (TEM) confirmed that the EV fraction contained intact EVs with the characteristic morphology and correct size range (**Figure 1D**). NTA revealed an average EV concentration of 2.0 ± 0.8×10^11^ particles/mL in the whole plasma samples before EVs isolation (**Figure 2A**). Following SEC-based isolation from 0.5 mL of plasma, EVs were precipitated and resuspended in 0.25 mL of filtered phosphate-buffered saline, yielding a final concentration of 3.9 ± 1.3×10^11^ particles/mL (**Figure 2B**). These results indicate recovery of 97.5% of plasma EVs. In comparison, the combined protein fractions #7–13 obtained by SEC contained a negligible number of EVs (3.7 ± 2.6×10^5^ particles/mL) (**Figure 2C**).

### Analysis of miRNAs from plasma-derived EVs

**Figure 3:**
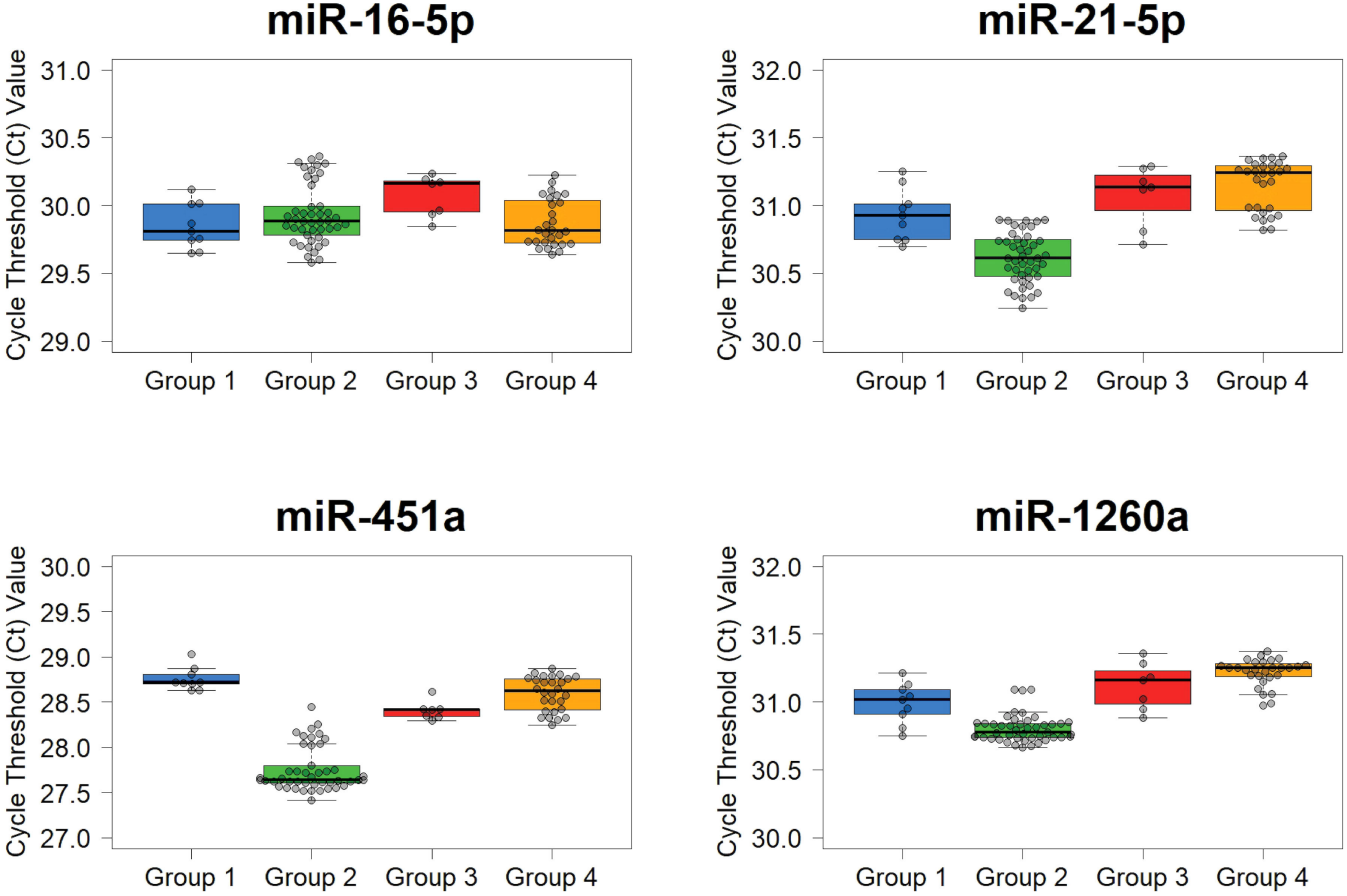
Identification of normalization controls. Cycle Threshold (Ct) Values of miRNAs exhibited the most stable expression across all patient groups. Among them, miR-16-5p and miR-1260a demonstrated the highest expression stability.

**Figure 4:**
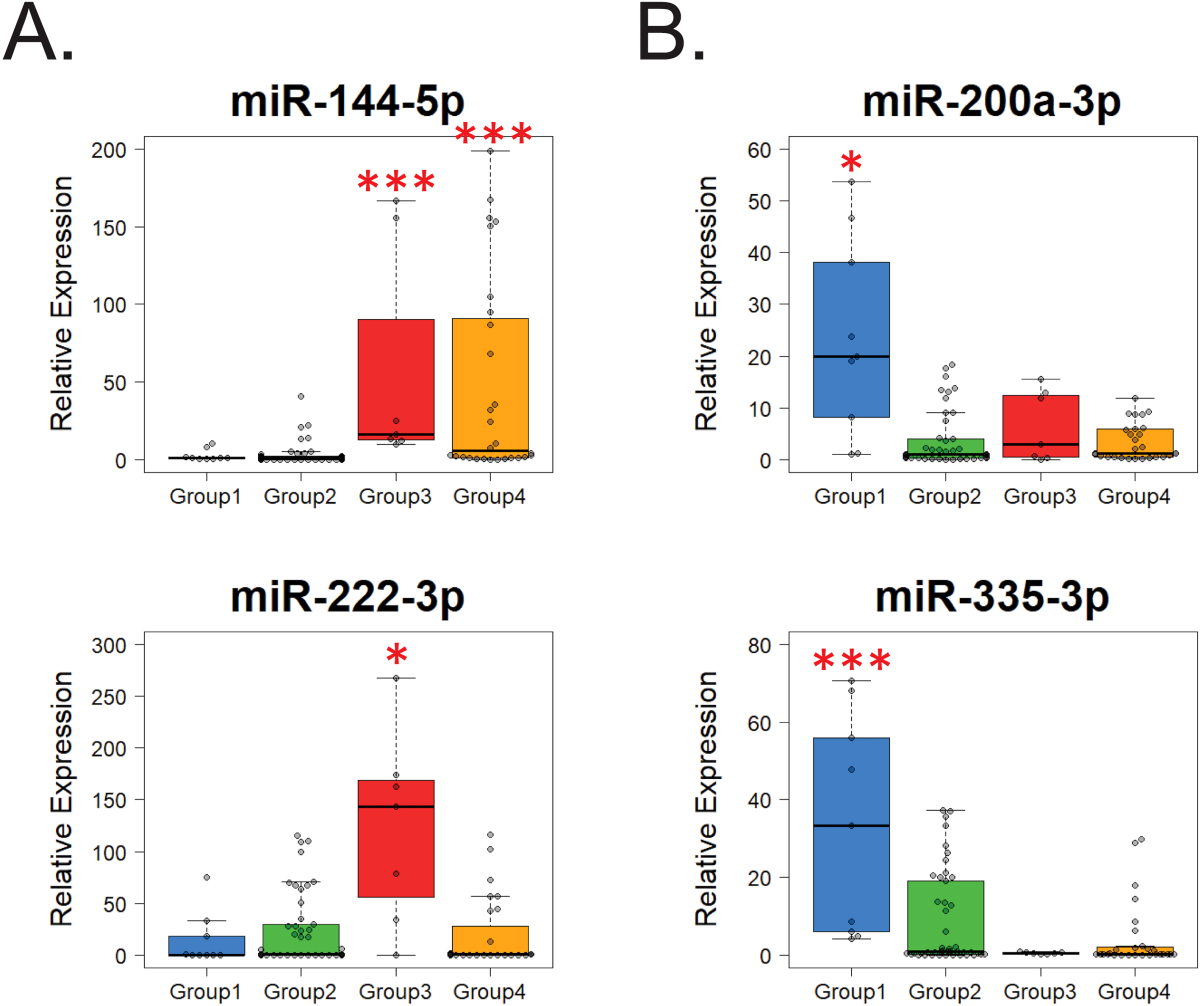
Relative miRNAs expression levels extracted from plasma EVs. Values were calculated using the 2^−ΔΔCt method. EV-associated miRNAs were analyzed from the plasma of the breast cancer patients divided into four groups: Group 1 – minimal toxicity, Group 2 - moderate toxicity, Group 3 - acute-high toxicity, and Group 4 - late-high toxicity. **(A)** miRNAs demonstrated significant increase of the relative expression in Group 3 (acute high post-RT toxicity) and Group 4 (late high post-RT toxicity). **(B)** miRNAs demonstrated significant increase of the relative expression in Group 1 (low/no post-RT toxicity). The P-values are shown as *p < 0.05 and ***p < 0.0001.

The group-based microarray analysis identified 164 miRNAs, 4 of which showed the most stable expression and were identified as potential normalization controls: miR-16-5p, miR-21-5p, miR-451a, and miR-1260a (**Figure 3**). Analysis of these miRNAs in all individual samples revealed that the most stable expression was demonstrated by miR-16-5p and miR-1260a (**Figure 3**). For further qPCR data analysis, miR-16-5p was used as a normalization control. Out of 164 analyzed miRNAs, 22 showed at least a 50-fold difference in relative expression between at least two groups. These 22 miRNAs were followed up with individual-level measurement via qPCR. Analysis of individual samples showed no statistically significant difference between groups of patients for 18 out of the remaining 22 miRNAs (**Supplementary Figure 2**). As presented in **Figure 4**, 4 of 22 miRNAs (miR-144-5p, miR-200a-3p, miR-222-3p, and miR-335-3p) demonstrated statistically significant differential expression across the analyzed groups.

Two miRNAs exhibited significantly elevated expression in the acute and late high post-radiotherapy (post-RT) toxicity groups (Groups 3 and 4) (**Figure 4A**). Specifically, miR-144-5p showed markedly higher relative expression in Group 3 (77.61 (12.76-90.38), *p* < 0.0001) and Group 4 (87.64 (1.11-88.75), *p* < 0.0001) compared with Group 1[(1.14 (0.61-1.74)] and Group 2 [2.28 (0.17-2.45)]. MiR-222-3p was significantly overexpressed exclusively in the acute high post-RT toxicity group [Group 3; 112.09 (56.21-168.29), *p* < 0.05] relative to Group 1 [17.63 (0.2-17.83)], Group 2 [29.23 (0.22-29.45)], and Group 4 [20.01 (0.11-20.12)]. In contrast, two miRNAs were significantly upregulated in the minimal toxicity group (Group 1) (**Figure 4B**). MiR-200a-3p demonstrated higher relative expression in Group 1 [29.94 (8.2-38.14), *p* < 0.05] compared with Group 2 [3.62 (0.33-3.92)], Group 3 [11.89 (0.5-12.39)], and Group 4 [5.33 (0.46-5.79)]. Similarly, miR-335-3p expression was significantly increased in Group 1 [49.94 (6.01-55.95), *p* < 0.0001] relative to Group 2 [18.95 (0.26-19.2)], Group 3 [0.34 (0.26-0.6)], and Group 4 [1.82 (0.15-1.97)].

## Discussion

NTA demonstrated that the isolated particles exhibited a mean diameter consistent with small EVs. Based on the SEC isolation protocol and the observed particle size distribution, the isolated vesicles are presumed to be predominantly exosomes. However, because NTA does not distinguish EV subtypes based on surface marker expression or other defining characteristics, definitive vesicle subtype identification cannot be established using this technique alone (24). Although the particle size and concentration observed in the present study are comparable to those reported previously, considerable variability exists across the literature, likely reflecting differences in sample type (plasma versus serum), patient populations, EV isolation methods, and particle characterization techniques (25–30). The combination of SEC and the PureExo isolation kit used in this study yielded a high-purity EV preparation suitable for downstream miRNA analysis and recovered 97.5% of total plasma EVs, supporting the robustness of this workflow for biomarker studies (**Figure 2**).

Several previous studies have identified specific miRNAs as suitable endogenous controls for normalization of miRNA expression data. In particular, let-7a-5p, miR-21-5p, and miR-16-5p have been reported to exhibit stable expression across healthy, benign, and malignant tissues (31). In the present study, miR-16-5p demonstrated the highest expression stability in plasma EVs and was therefore selected as the endogenous control, consistent with previous reports supporting its suitability for studies of metastatic breast cancer (31). The use of stable reference miRNAs minimizes biological and technical variability and improves the reliability of quantitative expression analyses (32).

This study identified two EV-associated miRNAs that may serve as candidate biomarkers associated with increased susceptibility to radiation-induced normal tissue toxicity in breast cancer patients. EV-associated miR-222-3p was upregulated exclusively in patients who developed acute high-grade toxicity, whereas miR-144-5p exhibited increased expression in both acute and late high-toxicity groups.

Breast cancer is a heterogeneous disease comprising multiple molecular subtypes with distinct biological characteristics, signaling pathways, and clinical behavior. However, the primary objective of the present study was to identify circulating EV-associated miRNAs associated with radiation-induced normal tissue toxicity rather than tumor biology or tumor response to therapy. Because all patients underwent surgical resection of the primary tumor before receiving adjuvant radiotherapy, the biological endpoint evaluated primarily reflects host and normal tissue responses to radiation exposure. To minimize potential confounding, clinicopathological variables, including molecular subtype, tumor grade, and disease stage, were incorporated into the statistical analyses. None of these variables was significantly associated with the candidate miRNAs, suggesting that the observed relationships are more likely attributable to individual radiosensitivity than to differences in tumor characteristics. Nevertheless, systemic effects associated with the underlying malignancy cannot be completely excluded, and validation in larger independent cohorts is warranted.

Notably, both miR-144-5p and miR-222-3p have been reported or are predicted to target manganese superoxide dismutase (SOD2), also known as manganese-dependent superoxide dismutase (MnSOD), a key mitochondrial antioxidant enzyme responsible for detoxification of reactive oxygen species (ROS). SOD2 plays a critical role in protecting normal tissues against oxidative stress induced by ionizing radiation and inflammatory cytokines (33, 34). Dysregulation of these miRNAs may therefore contribute to impaired antioxidant defenses and increased susceptibility to radiation-induced normal tissue injury. However, in our study the analyzed miRNAs were isolated from the total plasma EV population, neither their cellular origin nor their target cell populations can be determined from the present data. Furthermore, individual miRNAs regulate numerous target genes in a highly context-dependent manner, and computational target prediction alone provides only limited mechanistic insight without experimental validation. Future studies employing cell type-specific EV isolation, functional perturbation experiments, and *in vivo* models will be required to define the biological pathways through which these candidate biomarkers contribute to radiation-induced normal tissue responses.

An additional finding of this study was the identification of EV-associated miR-222-3p and miR-335-3p in patients who experienced no or minimal tissue toxicity following radiotherapy. Although this subgroup was relatively small, the observed differential expression may reflect biological mechanisms that promote tissue tolerance or adaptive responses to radiation-induced injury. Validation in larger independent cohorts will be required to confirm these associations and clarify their functional significance.

To our knowledge, this study is among the first to identify plasma EV-associated miRNAs associated with both acute and late radiation-induced normal tissue toxicity in breast cancer patients using a standardized SEC-based EV isolation workflow. These findings highlight the potential of circulating EV-associated miRNAs as minimally invasive biomarkers for individualized assessment of radiosensitivity.

The clinical significance of these findings lies in the potential to improve prediction of radiation-induced toxicity before treatment initiation. Unlike intratumoral miRNA analyses, which require invasive tissue sampling and may be influenced by intratumoral heterogeneity, circulating EV-associated miRNAs can be assessed through a minimally invasive liquid biopsy approach and may provide a more comprehensive representation of systemic responses to radiation exposure. Patients identified as having a high risk of severe radiotoxicity could potentially benefit from individualized treatment strategies, including optimization of treatment planning, implementation of advanced radiotherapy techniques, or early supportive interventions aimed at reducing treatment-related complications. In the future, therapeutic modulation of specific miRNAs may represent an additional strategy to enhance tumor radiosensitivity while protecting normal tissues from radiation-induced injury. Beyond breast cancer, these findings may also have broader implications for biomarker development in other malignancies and in individuals exposed to ionizing radiation under non-clinical conditions, such as occupational or accidental exposure (35).

To date, few studies are directly comparable to the present investigation. Existing studies examining miRNA biomarkers associated with radiotherapy-induced toxicity in breast cancer patients frequently employ different EV isolation methods, analytical platforms, patient populations, and toxicity assessment criteria, limiting direct comparison of findings (7, 21, 36). Furthermore, there is currently no consensus regarding the optimal endogenous miRNAs for normalization, which remains a significant challenge for reproducibility and cross-study validation (36). Standardization of EV isolation, normalization strategies, and analytical workflows will be essential for successful clinical translation of EV-associated miRNA biomarkers.

Overall, the present findings support the potential of plasma EV-associated miRNAs as minimally invasive biomarkers for predicting susceptibility to radiation-induced normal tissue toxicity. Validation in larger prospective cohorts and mechanistic studies investigating the biological functions of these miRNAs will be essential to confirm their clinical utility. Successful translation of these biomarkers into clinical practice could facilitate personalized radiotherapy planning, improve patient stratification, and ultimately reduce treatment-related toxicity while preserving the therapeutic efficacy of radiation treatment.

## Conclusion

This study identifies plasma extracellular vesicle-associated miRNAs as potential minimally invasive biomarkers associated with radiation-induced normal tissue toxicity in breast cancer patients. Using a standardized EV isolation workflow and quantitative miRNA analysis, we identified distinct EV-miRNA signatures associated with acute and late radiotherapy-related toxicity profiles. Specifically, increased expression of EV-associated miR-222-3p was observed in patients who developed acute high-grade toxicity, whereas miR-144-5p was associated with both acute and late high-toxicity groups. These findings suggest that circulating EV-associated miRNAs may reflect individual differences in radiosensitivity and may provide a foundation for improved risk stratification of patients undergoing radiotherapy.

The present study was designed to identify candidate biomarkers rather than define the complete biological mechanisms underlying these associations. Because the analyzed miRNAs were derived from total plasma EV populations, further studies are required to determine their cellular origin, functional targets, and contribution to radiation-induced tissue responses. Validation in larger, independent cohorts using standardized EV isolation and analytical approaches will be essential to establish clinical utility. Ultimately, integration of EV-associated miRNA biomarkers into precision radiotherapy strategies may enable individualized toxicity risk assessment, optimization of treatment planning, and reduction of radiation-induced normal tissue complications while maintaining therapeutic efficacy.

## Data Availability

The raw data supporting the conclusions of this article will be made available by the authors, without undue reservation.
