# Supplementary material for: Circulating extracellular vesicle miRNA as a novel predictive marker of radiation toxicity in breast cancer patients
Source: Front Oncol. 2026 Aug 5;16:1904861. doi: 10.3389/fonc.2026.1904861 (PMC13485556; doi:10.3389/fonc.2026.1904861)
Supplement: Supplementary file 1 [file DataSheet1.pdf]

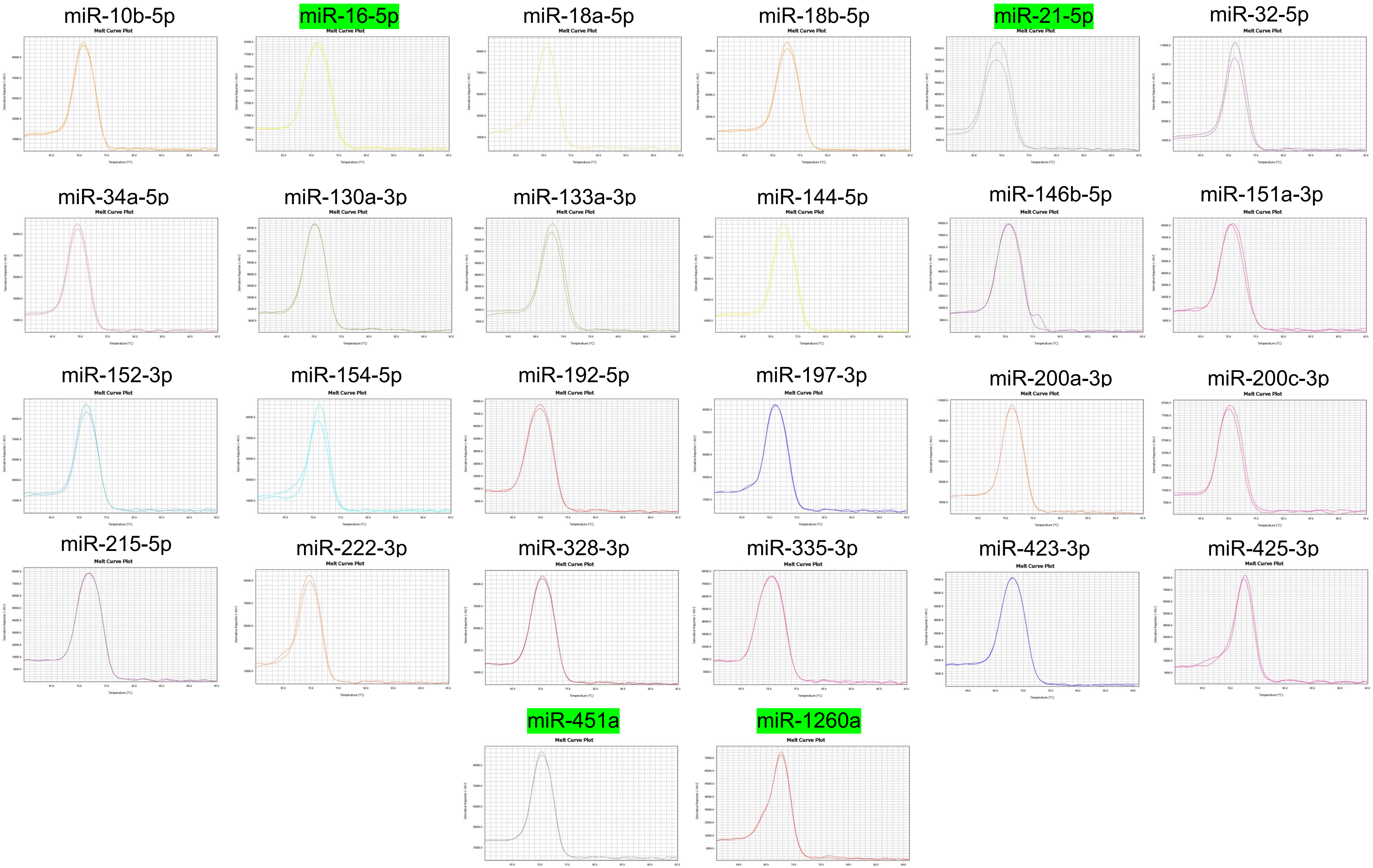

Figure S1. Melting curves for miRNAs extracted from plasma EVs of the breast cancer patients. 4 potential normalization controls (green) and 22 miRNAs followed up with individual-level measurement via qPCR.
