# Supplementary material for: Circulating extracellular vesicle miRNA as a novel predictive marker of radiation toxicity in breast cancer patients
Source: Front Oncol. 2026 Aug 5;16:1904861. doi: 10.3389/fonc.2026.1904861 (PMC13485556; doi:10.3389/fonc.2026.1904861)
Supplement: Supplementary file 2 [file DataSheet2.pdf]

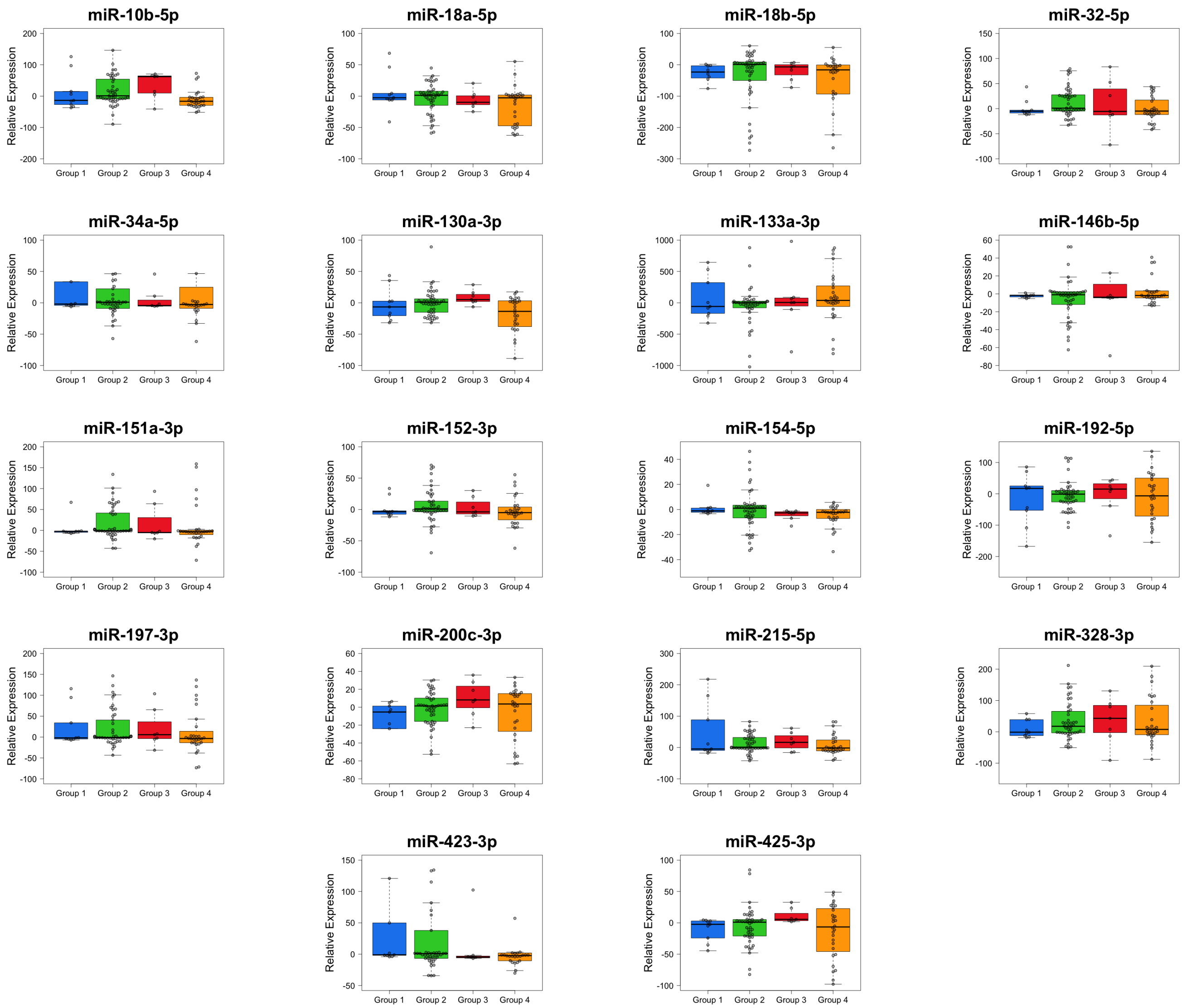

Figure S2. qPCR analysis of miRNAs extracted from plasma EVs. EV-extracted miRNAs with no statistically significant difference between groups of patients
