# Supplementary material for: Circulating extracellular vesicle miRNA as a novel predictive marker of radiation toxicity in breast cancer patients
Source: Front Oncol. 2026 Aug 5;16:1904861. doi: 10.3389/fonc.2026.1904861 (PMC13485556; doi:10.3389/fonc.2026.1904861)
Supplement: Supplementary file 3 [file DataSheet3.zip › Western Blot and TEM images.docx]

**Western Blot and TEM images:**

Representative WB images are shown in three different formats for each protein:

1. ‘Color’ – Dual-channel pseudocolor format, with the 700-nm (red) and 800-nm (green) fluorescence detection channels.
2. ‘Gray’ – The 800-nm near-infrared fluorescence channel was visualized in grayscale to facilitate assessment of individual protein bands intensity, with pixel brightness proportional to the detected fluorescence signal.
3. ‘Color high 700’ – The Dual-channel pseudocolor format. To improve visualization of membrane boundaries, the display intensity of the 700-nm channel was increased to produce partial signal saturation.

File ‘TEM_80kV 80kX 35886.tif’ contains original unmodified TEM image.
